# Supplementary material for: Smchd1 is a maternal effect gene required for genomic imprinting
Source: eLife. 2020 Nov 13;9:e55529. doi: 10.7554/eLife.55529 (PMC7665889; doi:10.7554/eLife.55529)
Supplement: Transparent reporting form [file elife-55529-transrepform.docx]

***eLife’s* transparent reporting form**

We encourage authors to provide detailed information *within their submission* to facilitate the interpretation and replication of experiments. Authors can upload supporting documentation to indicate the use of appropriate reporting guidelines for health-related research (see [EQUATOR Network](http://www.equator-network.org/%20)), life science research (see the [BioSharing Information Resource](https://biosharing.org/)), or the [ARRIVE guidelines](http://www.plosbiology.org/article/info:doi/10.1371/journal.pbio.1000412) for reporting work involving animal research. Where applicable, authors should refer to any relevant reporting standards documents in this form.

If you have any questions, please consult our Journal Policies and/or contact us: [editorial@elifesciences.org](mailto:editorial@elifesciences.org).

**Sample-size estimation**

- You should state whether an appropriate sample size was computed when the study was being designed
- You should state the statistical method of sample size computation and any required assumptions
- If no explicit power analysis was used, you should describe how you decided what sample (replicate) size (number) to use

Please outline where this information can be found within the submission (e.g., sections or figure legends), or explain why this information doesn’t apply to your submission:

We did not use a power calculation or sample size computation for our study. Replicate numbers for genomic analyses of Smchd1 maternal deletion range from 4 to 7 per group. Sample sizes of about 3 are typical for these types of analyses (RNA-seq, RRBS). For similar studies of imprinting, Inoue et al. 2018 Genes and Development and Hanna et al. 2019 Genome Biology used n = 2-4 samples per group. Variability is expected to be low in homogeneous genotypes of a single sex (male F1s of pure inbred mice). For each group, we include samples from at least two distinct litters. We further add to the replication by including two distinct genetic constructs for maternal deletion (MMTV-Cre and Zp3-Cre), with their respective controls.

For maternal and zygotic deletion experiments, we increased the replication to n = 6-13 because of the genetic heterogeneity (F1BC1 samples) meaning that on average only half of the genomic loci have SNPs that distinguish alleles.

ChIP-seq was performed in triplicates for NSCs, as is typical. For ChIP-seq in placenta we had only one replicate, however this sample was produced from three pooled placentae.

**Replicates**

- You should report how often each experiment was performed
- You should include a definition of biological versus technical replication
- The data obtained should be provided and sufficient information should be provided to indicate the number of independent biological and/or technical replicates
- If you encountered any outliers, you should describe how these were handled
- Criteria for exclusion/inclusion of data should be clearly stated
- High-throughput sequence data should be uploaded before submission, with a private link for reviewers provided (these are available from both GEO and ArrayExpress)

Please outline where this information can be found within the submission (e.g., sections or figure legends), or explain why this information doesn’t apply to your submission:

All replicate information is provided in the figure legends. All replicates are biological replicates rather than technical replicates as they represent different embryos. This is clear in the text, legends and methods.

Samples were only excluded from our analysis for the single preimplantation embryo sequencing where they were excluded based on well-defined quality control criteria, and for the Smchd1 heterozygous placental samples that had mosaic deletion based on PCR. These exclusions are described in the methods section.

All genomic data are available and the details are provided in the methods section.

**Statistical reporting**

- Statistical analysis methods should be described and justified
- Raw data should be presented in figures whenever informative to do so (typically when N per group is less than 10)
- For each experiment, you should identify the statistical tests used, exact values of N, definitions of center, methods of multiple test correction, and dispersion and precision measures (e.g., mean, median, SD, SEM, confidence intervals; and, for the major substantive results, a measure of effect size (e.g., Pearson's r, Cohen's d)
- Report exact p-values wherever possible alongside the summary statistics and 95% confidence intervals. These should be reported for all key questions and not only when the p-value is less than 0.05.

Please outline where this information can be found within the submission (e.g., sections or figure legends), or explain why this information doesn’t apply to your submission:

All statistical analyses are described in the methods and the test used is additionally mentioned in the figure legend. All exact p-values and additional details can be found in the supplementary tables.

(For large datasets, or papers with a very large number of statistical tests, you may upload a single table file with tests, Ns, etc., with reference to sections in the manuscript.)

**Group allocation**

- Indicate how samples were allocated into experimental groups (in the case of clinical studies, please specify allocation to treatment method); if randomization was used, please also state if restricted randomization was applied
- Indicate if masking was used during group allocation, data collection and/or data analysis

Please outline where this information can be found within the submission (e.g., sections or figure legends), or explain why this information doesn’t apply to your submission:

This is not relevant to our study as groups were assigned based on genotype.

**Additional data files (“source data”)**

- We encourage you to upload relevant additional data files, such as numerical data that are represented as a graph in a figure, or as a summary table
- Where provided, these should be in the most useful format, and they can be uploaded as “Source data” files linked to a main figure or table
- Include model definition files including the full list of parameters used
- Include code used for data analysis (e.g., R, MatLab)
- Avoid stating that data files are “available upon request”

Please indicate the figures or tables for which source data files have been provided:

All genomic data are available from the Short Read Archive under the BioProject accession PRJNA530651, with the details provided in the methods. We have provided a detailed description of our analysis for these data, which was performed in R and Seqmonk.

The allele-specific expression data is displayed in a raw form in the supplementary figures.
